# Challenges and opportunities for research clinicians interested in pain: results of a survey

**DOI:** 10.3389/fpain.2023.1194818

**Published:** 2023-08-24

**Authors:** Andrew Siddons, Laura Dover Wandner, Linda L. Porter

**Affiliations:** ^1^ N4 Solutions, LLC., Greenbelt, MD, United Stated; ^2^ National Institute of Neurological Disorders and Stroke, Office of Pain Policy and Planning, Bethesda, MD, United Stated

**Keywords:** pain, research training, workforce development, mentorship, clinical research

## Abstract

The National Institutes of Health and its independent advisors recognize the need to develop a strong pain research workforce and provide opportunities, particularly for clinicians, to pursue research careers. A survey was conducted to better understand the challenges facing the clinical pain research community. Respondents reported that time and funding to pursue research were the most critical factors either enabling or holding them back from a research career. Respondents who received some kind of formal research training or mentorship were more likely than those who did not to have federal research funding and to be at more advanced stages of their careers. The findings point to a need for all stakeholders in the pain research community to help formalize research training and provide funding or protected time to support the ambitions of aspiring researchers.

## 1. Introduction

In 2011, the Institute of Medicine issued a landmark report (1) on pain care in the United States, including a chapter devoted to research challenges. Among the points made by the report were that pain research needs more scientists from diverse disciplines and across basic, clinical, behavioral and social backgrounds. The authors recommended increasing the training of pain researchers, including through training grants from the National Institutes of Health (NIH), specifically advocating for pre- and postdoctoral fellows and junior investigators to promote pain research education.

In 2020, the Interagency Pain Research Coordinating Committee (IPRCC) discussed the promotion of a new generation of pain researchers at its November meeting (2).

Among IPRCC members' ideas for promoting a new generation of pain researchers was the suggestion to survey clinical and basic pain researchers to identify what factors could advance their interest in research rather than, for example, push them towards private practice.

The medical research community has been aware of challenges facing clinician-scientists for some time. In 2014, a working group convened by the NIH to make recommendations that could strengthen the physician-scientist workforce identified several challenges (3), including: the uncertainty of funding, the structure of training, debt, a poor work-life balance, a need for multiple mentors and pressure to increase institutional revenue through patient care.

The factors affecting participation and progression in clinical research range from personal (e.g., compensation, social capital, and confidence), orientation toward certain roles (e.g., preferences for administration, clinical care, and education) to interpersonal (e.g., mentorship and discriminatory behavior), to organizational (e.g., academic and clinical workplace culture) and policies (e.g., availability of mentors and active support for advancing careers) (4). There also can be challenges to finding a position that would help one gain clinical research experience without already having clinical research experience (5).

Some published literature has specifically examined the issue as it relates to pain research, but while pain management is a multidisciplinary field, the discourse on workforce development has primarily focused on anesthesiologists (6,7). One study (8) on the challenges facing the anesthesiology workforce notes that while protected time to develop research skills and conduct research is essential, the receipt of an NIH grant meant to support research training was rare. Other (9) obstacles that have been cited for pain researchers include financial challenges (financial disincentives to pursue research, as well as debt), the existence of adequate mentorship and the acquisition of research skills.

In response to the IPRCC's recommendation, the Office of Pain Policy and Planning (OPPP) within the National Institute of Neurological Disorders and Stroke (NINDS) developed a survey to examine the reasons why those with clinical degrees and an interest in pain might or might not embark on a career in clinical pain research.

We sought to expand on the currently published literature to include the broader multidisciplinary backgrounds that make up the clinical pain research workforce, which also includes nurses, dentists, psychologists, physical therapists and those from many other distinct medical fields.

## 2. Materials and methods

### 2.1. Sample

The survey population included pain clinicians or pain researchers across the career spectrum. Respondents could opt-in to taking the survey through non-specific invitations distributed across the pain research community through different pain management and research organizations: American Academy of Pain Medicine; American Psychological Association; American Society of Anesthesiologists; American Society of Regional Anesthesia and Pain Medicine; Foundation for Anesthesia Education and Research; Initiative on Methods, Measurement, and Pain Assessment in Clinical Trials(IMMPACT)/Analgesic, Anesthetic, and Addiction Clinical Trial Translations, Innovations, Opportunities, and Networks(ACTTION); Kaui'i Pain Conference; NIH Pain Consortium; Pain Research Forum; U.S. Association for the Study of Pain (USASP); and the Veterans Health Administration. There were no inclusion or exclusion criteria for participation in this survey.

### 2.2. Dissemination and data collection

The survey responses were collected in three waves. The first version of the survey was administered to attendees of the Kaua'i Pain Conference in March, 2021, using the Digitell platform (10). The second version of the survey was sent to members of the USASP. USASP leadership sent its members an invitation and link to the survey. The survey was accessible to anyone with access to the link. The final version of the survey was sent in a similar fashion to members of the aforementioned pain management and research organizations. Once again, anybody with access to the link was able to take the survey.

The survey was hosted on the SurveyMonkey platform. While SurveyMonkey prevents the same user from completing the survey multiple times if they use the same browser, and a question was included to help screen out respondents who had taken it previously, there was no way to ensure that there were not multiple responses from the same individual.

All survey responses were anonymous. The NINDS Office of Science Policy and Planning (OSPP) determined this initiative's activities did not qualify as research requiring protections for human participants and did not require Institutional Review Board (IRB) review.

### 2.3. Survey

The final version of the survey had 29 total questions, with respondents answering a minimum of 10 questions and a maximum of 22 questions. Two earlier versions of the survey had fewer questions.

For a full list of questions asked in the final version of the survey, see Table 1. The table notes which questions were asked in the initial version of the survey and the differences between the second and final versions of the survey.

**Table 1:** List of questions included in survey of clinical pain research workforce.

(1) What is your clinical background/degree? Select all that apply.^a^
(2) What is your specialty? Select all that apply.^a^
(3) How would you describe your primary work setting?^d^
(4) Have you ever been involved with clinical pain research?^a^
(5) Why did you decide to pursue a career in clinical pain research? Select all that apply.^d^
(6) If you are currently participating in clinical research, which of the following factors made it possible for you to do so? Select all that apply.^d^
(7) If you previously participated in clinical research, but no longer are, which of the following factors influenced your decision? Select all that apply.^b^
(8) If you plan to continue with, or would like to return to clinical research, what opportunities would help you stay or re-enter the field? Select all that apply.^a^
(9) Please feel free to use this space to elaborate on your answers about the factors influencing your decisions whether to pursue clinical pain research (no character limit).^d^
(10) If you have conducted clinical pain research, how would you broadly describe the area of focus? Select all that apply.^d^
(11) How would you best describe your stage of research experience?^a^
(12) What is or was your role in your research activity? Select all that apply.^a^
(13) How is or was your research activity funded? Select all that apply.^a^
(14) Have you received formal research training, including a structured syllabus, or mentoring during your career?^b^
(15) Was the training/mentoring specific to pain?^a^
(16) Did you ever receive any of the following NIH training or career development awards for your mentoring? Select all that apply.^a^
(17) If you received NIH funding, how did it impact your research training? Were there effects beyond the monetary contribution itself? (No character limit)^c^
(18) Did you provide formal training or mentoring in clinical research during your career to more junior clinicians or researchers?^a^
(19) Was the training or mentoring specific to pain?^a^
(20) Did your trainees continue in the clinical research field?^a^
(21) If you provided formal training or mentoring, did you have protected time to mentor provided through any of the following sources? Select all that apply.^a^
(22) If you have never participated in clinical pain research, have you considered it?^a^
(23) If you have considered going into clinical research but have never participated, which of the following factors affected your decision? Select all that apply.^d^
(24) If you have never considered participating in clinical research, what are the reasons why? Select all that apply.^b^
(25) What would help you participate in clinical research Select all that apply.^a^
(26) Please feel free to use this space to elaborate on the factors that have influenced your decisions regarding clinical pain research (no character limit).^d^

^a^ Included in version 1.

^b^ Modified from version 1.

^c^ Exclusive to version 3.

^d^ Only in versions 2 and 3.

The questions were developed by the NIH OPPP with feedback from the NINDS Office of Science Policy and Planning, and a group of experienced clinical pain investigators from outside of NIH.

### 2.4. Analysis

Responses were aggregated using Microsoft Excel and R, with overall response data and crosstabulations produced using R. The analysis was based on responses stratified by the following variables: whether the respondent had ever been involved in clinical pain research; stage of research experience; receipt of formal research training or mentoring; and providing formal research training or mentoring. Significance tests were done using two-proportion *Z*-tests conducted in Microsoft Excel.

## 3. Results

A total of 433 responses were collected: 105 from attendees of Kaua'i Pain Conference, 120 from the US Association for the Study of Pain, and 208 from the broader clinical pain and pain research community. The final analysis included 430 responses, after some incomplete responses were excluded from the data set. Many questions permitted respondents to select all answers that could apply, and for some questions, the authors are reporting only the top responses, so in those cases the percentages reported in this paper might not add up to 100%.

### 3.1. Respondent profile

Among the respondents (Table 2), the most common degrees were MD (41%), PhD (20%), clinical PhD (17%), RN (6%) and PsyD (5%). The most common specialties were pain (48%), psychology (26%) and anesthesiology (18%). The most common work settings were universities (22%), teaching institutions (21%) and government hospitals (19%).

**Table 2:** Characteristics of survey respondents by degree, work setting, pain research experience and funding.

Degree	*N*	% (of 430)
APRN	3	1
Clinical PhD	73	17
Chiropractor	4	1
DDS	9	2
DO	13	3
DPT	18	4
MBBS	3	1
MD	178	41
MPH	10	2
MS	18	4
OT	0	0.00
NP	8	2
PA	0	0.00
PharmD	8	2
PhD	88	20
PsyD	23	5
RN	27	6
Other	28	7
Specialty	*N*	% (of 430)
Acupuncture	3	1
Addiction medicine	10	2
Anesthesiology	76	18
Chiropractic	4	1
Clinical care	17	4
Critical care	4	1
Emergency medicine	3	1
Geriatrics	7	2
Gastroenterology	2	<1
Immunology	2	<1
Informatics	3	1
Primary care	25	6
Neurology	24	6
Neuroscience	19	4
OB/GYN	2	<1
Occupational therapy	0	0
Oncology	4	1
Pain	205	48
Pediatrics	19	4
Psychiatry	5	1
Psychology	112	26
Physical medicine and rehabilitation	15	3
Physical therapy	21	5
Rheumatology	9	2
Social work	2	<1
Surgery	6	1
Other	59	14
Work setting	*N*	% (of 430)
Clinic—community/public	20	5
Clinic—private	18	4
Hospital—government	82	19
Hospital—private	18	4
Hospital—university	52	12
Teaching institution	90	21
Non-profit organization	19	4
Pharmaceutical company	4	1
University	95	22
Other	21	5
Clinical pain research history	*N*	% (of 430)
Currently involved	206	48
Previously involved	94	22
Never involved	118	28
Research experience level	*N*	% (of 300)
Early-stage investigator (no mentor)	39	13
Early-stage investigator (mentored)	102	34
Transitioning to independent status	32	11
Established (independent)	110	37
Received formal research training or mentoring	*N*	% (of 300)
Yes	210	70
No	69	23
Missing	21	7
Was the training/mentoring specific for pain?	*N*	% (of 210)
Yes	70	33
No	80	38
Yes, in part	57	27
Missing	3	1
Provided formal research training or mentoring	*N*	% (of 300)
Yes	151	50
No	120	40
Missing	29	10
How is or was your research activity funded?	*N*	% (of 300)
Federal funding	166	55
State funding	15	5
Institutional funding	96	32
Departmental funding	67	22
Pharmaceutical or other industry funding	49	16
Patient grants	4	1
Start-up funds	18	6
Other private funding	84	28
No funding	51	17
Other	17	6

Most respondents reported some involvement with clinical pain research: 48% reported current involvement with clinical pain research, 22% were previously involved with clinical pain research, and 28% were never involved in clinical pain research.

Among those with research experience (*n* = 300), 37% were established, independent investigators; 11% were transitioning to independent status; 34% were early investigators with mentors, and 13% were early investigators with no mentor. Most respondents who identified as researchers (70%) received formal research training or mentoring, with a further 60% of those respondents reporting that the training or mentoring was related to pain.

Among the different work settings, a significantly larger proportion of respondents were engaged in research at teaching institutions compared to the overall sample: 31% to 21%, *z* = 2.75, *p* = .006) or universities (50% to 34%, *z* = 3.87, *p* < .001) were engaged in research when compared to the overall sample.

### 3.2. Funding for research

Federal dollars were the most common source of research funding, with 55% of those with research experience (*n* = 300) receiving federal funding. The next most common sources were institutional (32%) and other private funding (28%). Among those with research experience, 28% of respondents saying that they had received an NIH award meant for early-career researchers, mentored research projects or pre- and post-doctoral training.

### 3.3. Motivation for research

In response to the question, “Why did you decide to pursue a career in clinical pain research,” the top responses among all respondents who had ever been involved in research were: helping patients, families and other providers (69%); the pursuit of knowledge (69%); applying clinical skills to research (59%) and discovering new treatments (47%). However, there were differences between current researchers and former researchers in this regard (Table 3). Pursuit of knowledge was a reason for 75% of current researchers, compared to 54% of former researchers, *z* = 3.22, *p* = 0.001. While 77% of current researchers said that “helping patients, families and other providers” was a factor, just 49% of former researchers included this as a factor, *z* = 4.29, *p* < 0.001.

**Table 3:** Differences between current researchers and former researchers.

	Current researchers (*N*)	% (of 186)	Former researchers (*N*)	% (of 68)	*Z*-score	*P*-Value
Reasons for pursuing a career in clinical pain research						
Helping patients, families and other providers	143	77	33	49	4.29	<0.001
Pursuit of knowledge	139	75	37	54	3.22	0.001
Applying clinical skills to research	117	63	33	49	2.01	<0.001
Discovering new treatments	96	52	23	34	2.54	<0.001
Factors that would help clinicians remain researchers or return to research	*N*	% (of 206)	*N*	% (of 94)		
Resources for protected time	142	69	46	49	3.32	<0.001
NIH or other government support	148	72	35	37	5.77	<0.001
Institutional support	130	63	40	43	3.24	0.001
Career stage						
Early-career researcher	82	40	50	53	2.1	0.035
Established researcher	86	42	23	24	3.01	0.002
Received formal training or mentoring	154	74	57	61	2.27	0.023
Received NIH award for training/mentoring	71	35	13	14	3.74	<0.001

### 3.4. Factors influencing clinical pain research careers

Asked about factors that made it possible to conduct clinical pain research, the most common responses among current researchers (*n* = 206) were support/funding from institution/department (53%); protected time to develop grant applications (40%); and support from families and social networks (35%). Just 21% reported that funding from NIH was a factor.

Those who no longer participate in clinical pain research (*n* = 94) most commonly reported a lack of time to prepare grant applications (34%) as a factor influencing their decision to quit research. Other factors included limited funding opportunities (29%), complexity associated with clinical trials (19%) and undesirable work/life balance (17%).

The most common factors reported that would help clinicians remain researchers or return to research (*n* = 300) were resources for protected time (63%), NIH or other government support (61%) and institutional support (57%). For any of these factors, however, those who were former researchers (*n* = 94) were less likely than current researchers (*n* = 206) to say that any of these factors would be helpful (Table 3): 69% of current researchers said that resources to support protected time would be helpful, vs. 49% of former researchers, *z* = 3.32, *p* < 0.001; 72% of current researchers said NIH or other government support vs. 37 percent of former researchers, *z* = 5.77, *p* < .001; and 63% of current researchers said institutional support vs. 43% of former researchers, *z* = 3.24, *p* = .001.

For those who were never researchers, but have considered it (*n* = 68), 51% said that a deciding factor was a lack of protected time, followed by a lack of time to prepare grant applications (40%), lack of opportunities (32%) and lack of adequate training (31%).

### 3.5. Early-career challenges

Among former researchers (*n* = 94), 53% said they were early in their careers (Table 3), while 40% of current researchers (*n* = 206) were early in their career, *z* = 2.1, *p* = 0.035 In contrast, 24% of former researchers are established compared to 42% of current researchers that are established, *z* = 3.01, *p* = 0.002. Established researchers (*n* = 110) were also be more likely than early-career researchers (*n* = 141) to receive federal funding (Table 4): 76% of established researchers reported receiving federal funding, compared to 45% of early-career researchers, *z* = 4.94, *p* < 0.001.

**Table 4:** Differences between early-career researchers and established researchers.

	Early-career researchers—*N*	% (of 141)	Established researchers—*N*	% (of 110)	*Z*-score	*P*-value
Factors that made it possible to conduct clinical pain research						
Support/funding from institution/department	47	33	46	42	1.47	0.14
Protected time to develop grant applications	34	24	35	32	1.41	0.16
Support from families and social networks	25	18	29	26	1.53	0.13
NIH funding	9	6	28	25	4.26	<0.001
Factors that would help clinicians remain researchers or return to research						
Resources for protected time	97	68	72	65	0.50	0.617
NIH or other government support	81	57	80	73	2.62	0.009
Institutional support	96	68	75	68	0	1
Received federal funding for their research	63	45	84	76	4.94	<0.001
Received formal training or mentoring	92	65	90	82	2.99	0.003

The importance of the aforementioned factors that help enable research varied based on career stage (Table 4). Established researchers were more likely than early career researchers to say that funding from their institution (42% vs. 33%, *z* = 1.47, *p* = 0.14), protected time to develop applications (32% to 24%, *z* = 1.41, *p* = 0.16), or funding from NIH (25% to 6%, *z* = 4.26, *p* < .001) were factors that made it possible for them to pursue clinical research.

Some factors that would help established researchers (*n* = 110) continue their research careers were also less likely to be factors that would help early-stage researchers (*n* = 94) continue their research. Notably, while 73% of established researchers said NIH or other government support would continue their research careers, only 57% of early-career researchers said NIH funding would help them continue their careers, *z* = 2.62, *p* = .009.

### 3.6. Importance of training and mentoring

Among all who have ever engaged in research (*n* = 300), 70% have had formal training or mentoring. Among those who had received formal training or mentoring (*n* = 210), 33% said it was specific to pain; 27% said it was related to pain “in part” and 38% said it was not specific to pain (Table 1). But current researchers (*n* = 206) were more likely than former researchers (*n* = 94) to have received research training or mentoring (74% vs. 61%, *z* = 2.28, *p* = .023, Table 3). Among established researchers (*n* = 110), 82% received research training or mentoring, and 65% of early researchers (*n* = 141) received research training or mentoring, *z* = 2.99, *p* = .003, Table 4.

Those who received formal research training or mentoring were also more likely to receive federal funding. While the percentage of all researcher respondents (*n* = 300) who received federal funding was 55%, 67% of those who received formal research training and mentoring (*n* = 210) received federal funding, compared to 39% of those who did not receive formal research training or mentoring (*n* = 69), *z* = 3.96, *p* < .001).

Those who were still in research (Table 3) were more likely to have received an NIH training or career development award: 34% of current researchers (*n* = 206) received an award, compared to 14% of former researchers (*n* = 94), *z* = 3.59, *p* < .001.

### 3.7. Characteristics of mentors

Among respondents who have ever been involved in research (*n* = 300), 50 percent have provided formal training or mentoring. The percentage is greater among current researchers (*n* = 206, 61%), but less among former researchers (*n* = 94, 44%, *z* = 2.55, *p* = .011). Among those who are established in their careers (*n* = 109, current and former researchers), 91% provided formal training or mentoring. Mentors (*n* = 151) were more likely than the overall sample (*n* = 430) to be working at teaching institutions (32% vs. 21%, *z* = 2.49, *p* = .013).

Among those who provided mentoring (*n* = 151), 85% said it was specific to pain. Asked if their trainees continued in the clinical research field, 24% said “most” (75% or more) continued; 30% said “some” (25% to 75%) continued; and 30% said “a few” (less than 25%) continued; and 7% said “none” continued.

While mentoring is fairly common, especially among more established researchers, few reported having funded protected time to support their mentoring activities. Among those who reported providing training or mentorship (*n* = 151), the most common sources of funding for protected time to mentor was from their departments (25%) or institutions (23%), followed by a federally-funded award (16%). But the most common answer was “no funding received” (49%).

## 4. Discussion

This was the first survey asking multidisciplinary research clinicians who specialize in pain to identify the factors that have helped them pursue clinical pain research as a career, or, conversely, held them back from pursuing research alongside their responsibilities in patient care.

The findings in this survey are similar to the findings of Hall (11), who wrote that time and lack of formal research training were the most likely barriers to neurologists hoping to pursue clinical research. In that study, it was found that the amount of time spent on research training may not be adequate for those who wish to conduct more complex or larger studies, which would affect the type of clinical research being done.

Based on the responses in this study, it could be inferred that those who attempted to pursue research careers but have returned to focus exclusively on patient care tend to be earlier in their careers (53% of former researchers were early in their careers, vs. 40% of current researchers, while 42% of current researchers are established vs. 24% of former researchers) and are less likely than current researchers to have received any formal research training or mentoring (among current researchers, 74% received training or mentoring, vs. 61% of former researchers).

Other workforce researchers also have found that quality mentorship and exposure to research during medical school or earlier have a positive effect on factors like efficacy—belief in one's own ability to pursue scientific research—that can inspire physicians to enter clinical research (12–15). In addition, a lack of specific training programs and mentorship (16) can be an obstacle.

This survey's findings also are compatible with the assertion by Adams and Memtsoudis (7) that the mentorship model, while valuable for many, may have drawbacks for some compared to more regular, formalized research training. Our findings showed that those who were still in research were more likely to received formal training and mentoring (74% of current researchers vs. 61% of former researchers); are more likely to have received federal research funding (67% of those who received training and mentoring vs. 39% among those who didn't); and that there may be a disparity between the training and mentoring that was available to those who are further along in their careers compared to those who are at the beginning of their careers today (82% of established researchers received training or mentoring vs. 65% of early researchers. That there was a portion of respondents that attempted research and have dropped out, or are interested in research but haven't pursued it, suggests that more formal research training is needed. Adams and Memtsoudis suggest that having a more reproducible research training infrastructure would be a greater benefit to more younger scientists and could help avoid issues such as mismatches between mentors and trainees or conflicts over prioritizing the mentor's own research over that of the trainee.

Across career stages, there are several factors that appear most important to help clinicians continue their careers with pain research: support and funding from their home institutions (53% considered important), and protected time to enable them to develop applications and conduct research (40% considered important). The finding regarding protected time echoes Meador's findings (17) on academic medicine more generally, with financial pressures seeing faculty increasing their clinical activities (which bring in money) at the expense of time to conduct research (which costs money).

Among our survey respondents, 28% reported receiving NIH awards for training or career development. This proportion is consistent with the 28% average success rate in recent years for applicants for NIH Fellowships (“F” awards), and is similar to the 33% average rate in recent years for NIH career development grants (“K” awards). It is lower, however, than the 52% average rate for NIH research training grants (“T” awards) (18).

Support from the NIH itself was more important for established investigators, who were more likely to have received NIH funding than those earlier in their careers (25% of established researchers considered it important, compared to 6% of early-career researchers). Previous publications on challenges facing clinical researchers have noted that it takes time for younger faculty members to support themselves through grants, suggesting the need for bridge awards until independence can be established (19). In combination, these findings from the literature and the present survey's findings suggest that research funders and institutions should consider the importance of providing protected research time across career stages if they want to promote a research environment that is accessible to a greater number of interested clinicians.

If there are efforts to attract former investigators back to research, those might pose a different kind of challenge. The survey found that those who have dropped out of research were significantly less likely to say that any of the most important factors that facilitate research (protected time, NIH funding, or institutional support) would be helpful to return to that career trajectory.

### 4.1. Limitations

As this was an anonymous survey, it is possible that respondents may have taken the survey multiple times, or that they may have been misrepresenting themselves in their responses. Similarly, since this was an anonymous survey, we do not have demographic data of the participants who completed it. Given that the survey was distributed through channels that tend to be more research-focused, the survey was also biased toward those who are already tapped into the research community in some way and may have biased the results towards those with interest and experience in research rather than those who are exclusively in clinical practice. The survey was administered approximately one year into the beginning of widespread SARS-Cov2 transmission in the U.S. With the numerous implications that restrictions to limit the spread of the virus had for both healthcare settings and other workplaces, and at people's own homes, it is likely that some of the responses reflected increased challenges and difficulties caused by the pandemic.

### 4.2. Implications/next steps

The results of the survey suggest that there are system level and individual level interventions that can help expand the clinical pain research workforce. In order to address these factors, however, a diverse group of stakeholders (e.g., funding organizations, universities and institutions, and research organizations) should consider how they can help enhance the workforce.

The NIH, one of the primary funding organizations within the United States, have already reviewed the survey results presented in this article and have begun to release Notice of Funding Opportunity (NOFO)s to address some of the concerns that clinical researchers reported in the survey. For example, in 2021 Midcareer Investigator Awards in Patient-Oriented Research (K24) (20), which are meant include support for mentoring, were awarded to HEAL clinical pain grantees so that they can devote additional time to mentoring earlier-stage members of their research teams. In 2022, a Clinical Scientist Institutional Career Development Award (K12) (21), grant was awarded to help promising pain researchers across the country access a rigorous research training program with mentors outside of their home institutions. Also in 2022, a grant was awarded to help enhance research infrastructure (R24) (22) established a pain research coordinating center to help increase the access to mentorship, training, and multidisciplinary collaboration among pain investigators.

However, the survey's findings suggest that the NIH alone cannot meet the needs of aspiring clinical pain researchers. There are numerous ways that the broader pain research community, including universities, hospitals and organizations representing researchers and practitioners, could contribute to enhancing the clinical research workforce.

Universities and institutions can play a role in enhancing the clinical pain research workforce. The survey's results suggest that a person is more likely to continue a career in research if they receive research training and mentoring in pain research. Thus, universities and institutions that have offer graduate training, fellowship, or post-doctoral training, in disciplines that treat pain to enhance their pain education, mentorship, and research training earlier in a person's career to help encourage a career in research.

Research organizations could also help enhance the pain research workforce, based on the results of this survey. Some research organizations are providing courses, training, mentoring to their early-stage investigators (ESI), as well as provide small research training grants to help initiate the careers of ESI. For example, the Foundation for Anesthesia Education and Research has several programs to promote younger investigators, including one to bring undergraduates into anesthesiology research labs, and grants to early-career clinicians that require their departments to provide protected time for research (8). Similarly, the U.S. Association for the Study of Pain provides either (1) the Rita Allen Foundation Award for chronic pain research to early-career leaders in basic pain research whose work has the potential to uncover new pathways to treat chronic pain, and (2) the MAYDAY Fund Award to support innovative projects to close the gap between knowledge and practice in the treatment of pain.

The survey suggests that when institutions provide protected time to their ESI, it is more likely that the researcher will stay in the research field. Thus, it may be helpful to provide more protected time for ESI as they are launching their careers. It could also be helpful to provide support to ESI as they are applying for their first federally supported grant, vs. requiring a professor to have a grant before they can be hired at a university, hospital, and/or institution.

### 4.3. Conclusion

There are many clinicians and medical professionals with an interest in treating pain who could help discover innovative and effective pain management techniques, but their ability to conduct the research to generate evidence in support of innovative pain management is hindered by many factors beyond their control, such as the need to prioritize day-to-day patient care, a lack of protected time to pursue research, and a challenging financial environment in terms of winning a research grant as well as institutional pressures. While additional research funding would help interested clinicians pursue research, funding alone won't fix other issues that hold aspiring researchers back, such as the need for a more formal research training paradigm. Creating a sustainable clinical pain research workforce will require a coordinated effort by research funders, training institutions, pain management professional organizations and other parts of the health care system.

## Data Availability

The datasets presented in this study can be found in online repositories. The names of the repository/repositories and accession number(s) can be found below: https://figshare.com/projects/Pain_research_workforce_survey/163009.
